# Controlling Amyloid Assembly Dynamics Using Spin Interfaces

**DOI:** 10.1021/acsnano.5c06285

**Published:** 2025-07-28

**Authors:** Yael Kapon, Dror Merhav, Gal Finkelstein-Zuta, Omer Blumen, Naomi Melamed-Book, Yael Levi-Kalisman, Ilya Torchinsky, Shira Yochelis, Daniel Sharon, Lech Tomasz Baczewski, Ehud Gazit, Yossi Paltiel

**Affiliations:** † Institute of Applied Physics, 26742 The Hebrew University, Jerusalem 9190401, Israel; ‡ The Shmunis School of Biomedicine and Cancer Research, George S. Wise Faculty of Life Sciences, 495190 Tel Aviv University, Tel Aviv 6997801, Israel; § Institute of Chemistry, Hebrew University of Jerusalem, Jerusalem 91904, Israel; ∥ Bio-Imaging Unit, The Alexander Silberman Institute of Life Science, The Hebrew University, Jerusalem 9190401, Israel; ⊥ The Harvey M. Krueger Family Center for Nanoscience and Nanotechnology, The Hebrew University, Jerusalem 9190401, Israel; # Institute of Physics, Polish Academy of Sciences, Warsaw 02668, Poland

**Keywords:** self-assembly, spin interactions, amyloids, Chiral-Induced Spin Selectivity (CISS), spin interfaces, spin-controlled assembly dynamics

## Abstract

Protein aggregation into amyloid fibrils is central to numerous diseases, yet the role of electron spin interactions during nucleation and self-assembly remains unexplored. We investigated amyloid formation of A-β(1–42) polypeptide, implicated in Alzheimer’s disease, and its smaller recognition motifs on ferromagnetic substrates. We observed a strong dependence of fibril formation dynamics on the substrate’s magnetization orientation using electron and fluorescence microscopy. Specifically, one magnetization orientation yielded approximately twice as many and significantly longer (up to 20-fold) fibrils compared with the opposite orientation, a preference that flipped with the opposite monomer chirality. Furthermore, ATR–FTIR detected structural variations in the fibril structure, depending on the substrate magnetization. These findings suggest that transient spin polarization of the monomers during self-assembly, potentially driven by the Chiral-Induced Spin Selectivity (CISS) effect, plays a critical role in amyloid assembly dynamics. The consistency of these effects across different molecule length scales suggests a fundamental spin-based influence on biomolecular aggregation. This insight may have implications for therapeutic strategies, including the use of spin-polarized magnetic nanoparticles to selectively modulate amyloid formation in neurodegenerative diseases and the integration of spin-selective interfaces in dialysis systems to mitigate dialysis-related amyloidosis.

## Graphical abstract

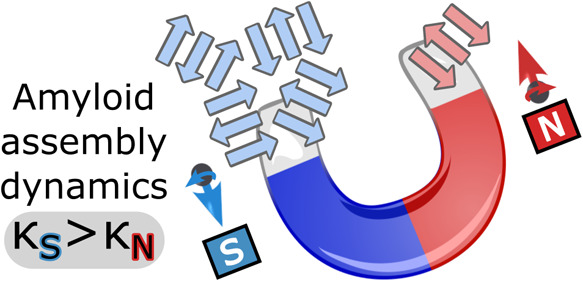

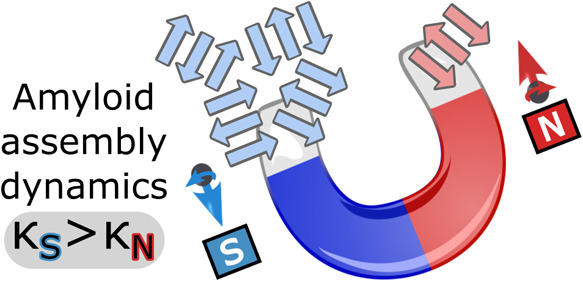

## Introduction

Amyloid fibrils are highly ordered protein aggregates implicated in numerous pathological conditions, including Alzheimer’s, Parkinson’s, and Type II Diabetes, where their formation disrupts normal cellular functions.^1^ At the same time, their highly ordered nanostructure has been explored as functional biomaterials in nanotechnology due to their unique biophysical properties.^2^

Even very short aromatic peptide fragments, including diphenylalanine peptides (Phe–Phe), representing the core recognition motif within the Amyloid β (Aβ) polypeptide, form well-ordered nanotubular assemblies with amyloid-like structural signatures.^3^ These phenylalanine residues (Phe19 and Phe20) are suggested to mediate intermolecular interactions between polypeptide chains.^3^ This hypothesis is supported by their role as key components in the formation of amyloid-like fibrils.^4,5^ Remarkably, even single phenylalanine amino acids can form similar amyloid-like structures,^6^ underscoring the fundamental role of aromatic interactions in self-assembly processes.^7^

The influence of external fields, such as electric and magnetic fields, on amyloid formation has been a topic of increasing interest.^8,9^ Due to the strong electric dipole and low diamagnetism of the monomers, these fields can modulate molecular interactions and orientation, thereby impacting the nucleation and growth of fibrils. Magnetic fields required for such effects tend to be extremely strong, and the low diamagnetism of peptides necessitates using magnetic fields exceeding 10 T for significant influence.^10^ In addition, using electric fields during the peptide self-assembly process can induce a uniform and controlled polarization. This effect however requires also a relatively high electric field of the order of 1 kV/mm.^11,12^

While these effects are relatively well-documented, the role of electron spin interactions remains largely unexplored in biological systems. Many biological molecules, including DNA,^13,14^ various proteins,^15–19^ and sugars,^20–22^ demonstrated significant spin-selective conductance due to the Chiral-Induced Spin Selectivity (CISS), a phenomenon relating molecular chirality to the electron spin.^23^ While it traditionally requires a current through the chiral molecule, recent advancements demonstrated that spin polarization could also appear due to transient charge displacement during adsorption on magnetic substrates. Achieving enantio-separation due to interactions with perpendicularly magnetized substrates.^24–26^ These spin-exchange interactions are influenced by the adsorption kinetics, with prolonged adsorption times leading to a loss of selectivity. Moreover, in a biological context, spin polarization was observed due to transient charge displacement during intermolecular interactions,^27–29^ opening possibilities for exploring spin-dependent phenomena in biological systems (Figure 1a).

**Figure 1:**
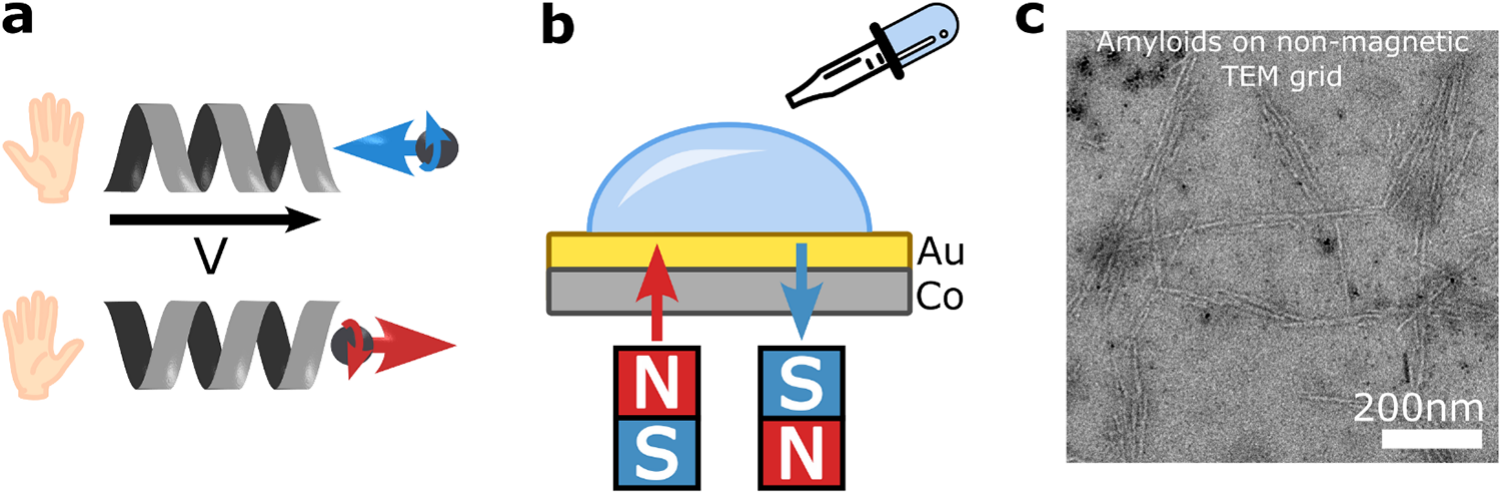
(a) Electron displacement through chiral molecules depends on their spin due to the Chiral-Induced Spin Selectivity (CISS) effect, leading to transient spin polarization in the molecule. (b) Experimental setup: A 40 μM solution of Aβ polypeptide in PBS was drop-casted onto an Al_2_O_3_(0001)/Pt­(5 nm)/Au­(20 nm)/Co­(1.3 nm)/Au­(50 nm) substrate, magnetized either North (red) or South (blue) in a perpendicular direction to the surface, incubated, and dried at 37 °C to allow amyloid-like fibril formation. (c) TEM image shows negatively stained fibrillar aggregates approximately 200 nm in length and 10 nm in diameter.

While spin polarization has been measured in short peptides and single amino acids, generally the longer the molecule and its electrical dipole, the larger its spin polarization.^13,30^ This makes growing systems, such as self-assembly or polymerization, particularly intriguing due to their evolving properties and potential accumulation of electric dipoles. For example, nucleation rates of conglomerate crystallization,^22^ electro-polymerization processes,^31,32^ and self-assembly processes^20,33^ were significantly different on differently magnetized substrates. In most cases, an amplification mechanism is needed to achieve effects over large time scales.

In the context of spin-controlled self-assembly, the aggregation of proteins into amyloid-like fibrils is particularly noteworthy for three reasons. First, intercepting nucleation events is critical for addressing amyloid-related diseases, and while functionalized surfaces have been employed for this purpose,^34^ magnetic substrates remain unexplored. Second, amyloids exhibit pronounced stereoselectivity,^35^ which may serve as an amplification mechanism for spin-dependent aggregation. Third, the Aβ polypeptide carries a significant charge, and its fibrils form a strong electrical dipole,^36^ further supporting their potential in spin-selective processes.

In this study, we demonstrate how spin interactions influence the aggregation of proteins into amyloid fibrils. By leveraging magnetic substrates, we establish that the amount of fibril formation is strongly influenced by substrate magnetization direction and monomer chirality, its size, and electrical dipole or polarization. Additional structural analysis reveals structural changes in the resulting fibrils due to substrate magnetization. These findings highlight the importance of transient spin polarization, mediated by the CISS effect, in self-assembly processes.

## Results

### Amyloid β (1–42) Aggregation on Magnetic Substrates

The effect of substrate magnetization direction on the amyloid-like aggregation of Amyloid β (1–42) (Aβ) polypeptide was studied by using the experimental setup illustrated in Figure 1b. A solution of Aβ 1–42 protein in Phosphate-buffered saline (PBS) was drop-casted onto Al_2_O_3_(0001)/Pt­(5 nm)/Au­(20 nm)/Co­(1.3 nm)/Au­(50 nm) molecular beam epitaxy (MBE) grown epitaxial nanostructure used as a substrate. The above nanostructures possess a perpendicular magnetic anisotropy (PMA), making them well-suited for magnetization reorientation by a short pulse of low magnetic field applied out of the plane, therefore avoiding induced undesired magnetic field effects on the assembly process. The substrates were magnetized in either a North (red) or South (blue) direction perpendicular to the plane by using a permanent magnet. The comparison between the two magnetization directions was conducted using a combination of confocal fluorescence microscopy with Thioflavin T (ThT) dye, scanning electron microscopy (SEM), and Transmission Electron Microscopy (TEM) to examine their quantity and morphology. The samples were dried at 37 °C on the magnetic substrate to allow amyloid fibril formation. Concurrently, the leftover protein solution was incubated at 37 °C for 24 h and dried on a transmission electron microscopy (TEM) grid for the structural analysis.

The TEM imaging of the negatively stained sample showed that the Aβ polypeptide self-assembled into fibrillar structures that were approximately 200 nm in length and 10 nm in diameter (Figure 1c). Further analysis of the structure using Attenuated Total Reflection Fourier Transform Infrared (FTIR) showed that the fibrillar structures are indeed Amyloid-like and composed of β sheets, as discussed further in the structural analysis section. Confocal fluorescence microscopy using ThT dye, excited at 405 nm and collected at 480 nm, was employed to detect and quantify the number of amyloid-like fibrils on substrates magnetized in either the North or South directions, respectively. ThT is a fluorescent dye commonly used in microscopy; it specifically binds to amyloid fibrils and enhances its fluorescence intensity. The fluorescence intensity is thus proportional to the total of amyloid-like structures in the studied sample (see Supporting Information for more details).

To ensure robust sampling, two independent samples were prepared for each magnetization direction, and three 635 μm × 635 μm regions per sample were analyzed, avoiding edges to minimize the effect of the coffee ring. The fluorescence intensity of each image was then taken and averaged between the different samples and regions. The baseline ThT fluorescence in PBS (48) was reduced from the average intensity. Given the 3 × 3 mm size of the drop-cast area, this approach allows imaging of most of the deposited sample. Obviously, the solution concentrations are the same for all measured samples. Quantification of average fluorescence intensity (Figure 2c) showed values of 109 ± 14 counts for South and 62 ± 10 counts for North magnetization orientation. This nearly 2-fold increase in intensity indicates higher amyloid aggregation for South-magnetized substrates and strongly supports that substrate magnetization influences the protein self-assembly process.

**Figure 2:**
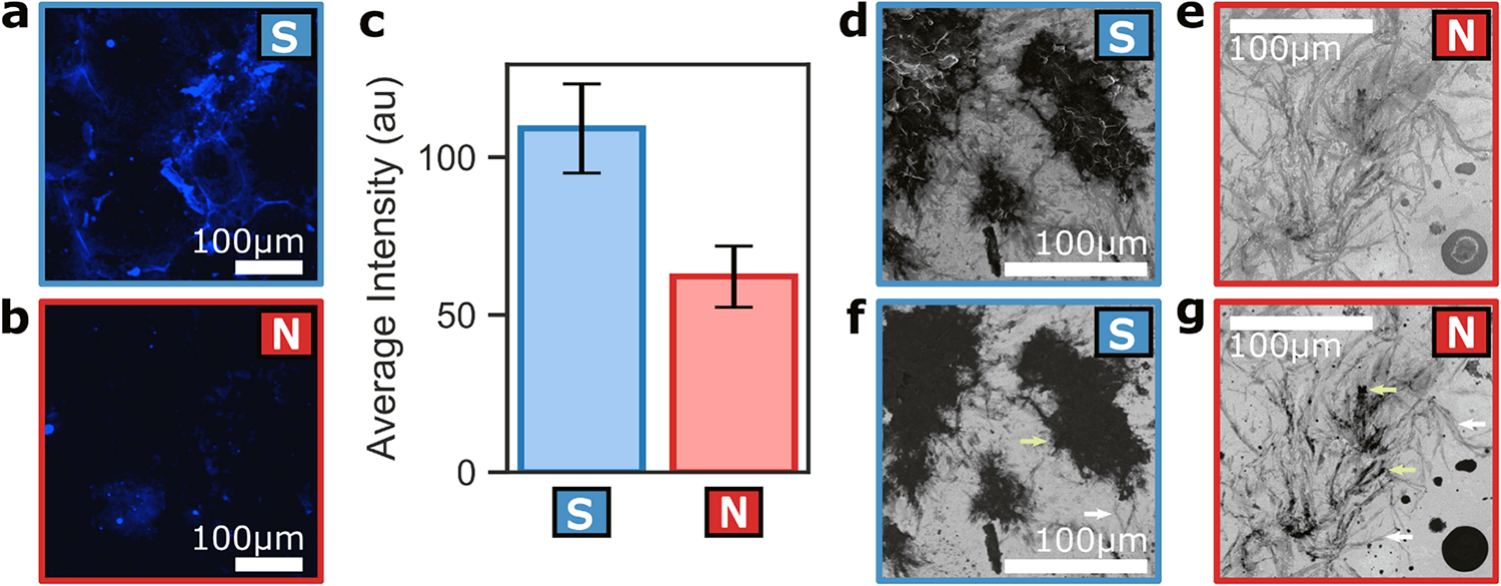
(a, b) Fluorescence microscopy images of amyloid-like fibrils stained with ThT, excited at 405 nm, and collected at 450–550 nm. South magnetization direction (a) exhibits increased amyloid formation compared to the North magnetization direction (b), as indicated by the higher fluorescence intensity. (c) Quantification of average fluorescence intensity shows significantly higher count values for South magnetization (157 ± 14) compared to the North direction (110 ± 10). Errors represent the standard error of the mean. (d–g) SEM secondary electron images (d, e) and corresponding backscattered electron images (f, g) reveal denser aggregation for the South magnetization direction (d, f), consistent with fluorescence results. Backscattered images (f, g) differentiate between the surface (Au – bright area), protein (dark, yellow arrows), and residual salt from the solution (midgray, white arrows).

SEM secondary electron images (Figure 2d, e) and corresponding SEM backscattered electron analysis (Figure 2f, g) provide insight into the morphology of amyloid aggregates formed on magnetic substrates. The SEM images reveal that the short fibrils observed in the TEM (Figure 1c) assemble into larger clumps on the substrate. However, the SEM technique does not resolve the fine details of individual fibrils visible in TEM, focusing instead on the larger-aggregation scale.

In backscattered electron images, intensity is directly related to the atomic number of the material, while higher atomic number elements appear brighter due to stronger electron scattering, but elements with lower atomic number appear darker. In the backscattered images (Figure 2f, g), the bright regions correspond to the Au cover layer of the substrate, the dark regions (marked in yellow arrows) to Aβ polypeptide aggregates, and the midgray regions (marked in white arrows) to residual salt from the solution. This analysis reveals that much of the material visible in SEM images for the North magnetization direction (Figure 2g) is salt, indicating that the amyloid aggregation itself is less dense than might initially appear. Consistent with the fluorescence microscopy results, the South-magnetized samples exhibit denser amyloid-like aggregation (Figure 2d, f) compared with North-magnetized samples (Figure 2e, g).

### Breaking Down the Protein - the Role of the Monomer’s Dipole Moment

We explored the role of monomer dipole moments in the spin-dependent self-assembly. The Aβ 1–42 polypeptide is fragmented into two smaller monomers, Phe–Phe dipeptide, and single amino acid, Phe, as illustrated in Figure 3a. The structure is taken from Lührs et al.^37^ Breaking down the protein reduces and shifts its overall dipole moment. Phe–Phe, a core Aβ recognition motif, is symmetric, and its dipole moment is aligned along the peptide backbone in the direction of fibril growth, unlike Phe, whose dipole is perpendicular, similar to the Aβ polypeptide (gray arrows, Figure 3a).

**Figure 3:**
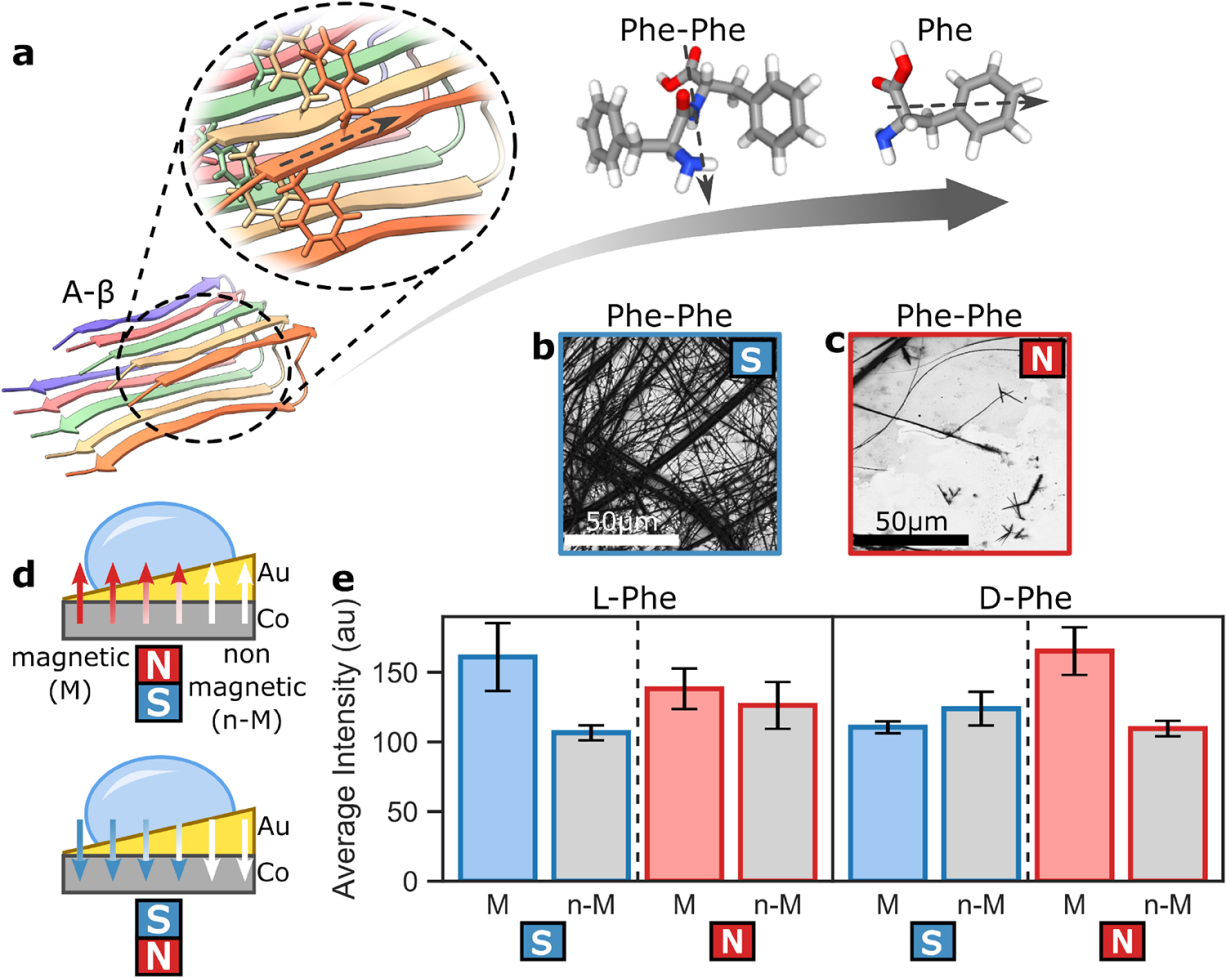
(a) Schematic representation of the Aβ protein (structure from PDB: 2BEG) and its breakdown into a Phe–Phe peptide and a single Phe amino acid (structures from PubChem). The direction of the dipole moment is marked with a dashed arrow. (b) SEM image of Phe–Phe fibrils formed on a South- (b) or North (c) magnetized substrate. Significantly longer fibrils formed on the South-magnetized sample. (d) Assembly of l-Phe or d-Phe amino acids drop-casted onto a magnetic substrate magnetized in the North (red) or South (blue) direction. Magnetic (M) and nonmagnetic (n-M) regions are created by varying the Au layer thickness on a Co substrate (Au cover layer was deposited as a wedge), allowing comparison of magnetic effects within the same drop. (e) Average fluorescence intensity for l-Phe shows higher values for the South-magnetized sample (M S = 161 ± 24) compared to the North-magnetized sample (M N = 139 ± 15) and nonmagnetic sides (n-M S = 107 ± 5, n-M N = 127 ± 17). For d-Phe, the North-magnetized sample (M N = 166 ± 17) exhibits higher intensity than the South-magnetized sample (M S = 111 ± 4), with comparable values for the nonmagnetic areas (n-M N = 110 ± 6 and n-M S = 124 ± 12). Error bars represent standard error of the mean.

Similarly to the Aβ polypeptide, Phe–Phe (0.1 mg/mL) was drop-casted onto magnetized substrates (Al_2_O_3_(0001)/Pt/Au/Co/Au) and left to self-assemble. SEM and ThT fluorescence microscopy quantified fibril morphology and aggregation. When formed on a South-magnetized substrate (Figure 3b), the Phe–Phe monomers formed long fibers extending beyond the area of view of the SEM imaging (greater than 250 μm). In contrast, when formed on a North-magnetized substrate (Figure 3c), the Phe–Phe monomers reveal a heterogeneous assembly consisting of long fibers (>250 μm) and significantly shorter fiber structures (approximately 10 μm).

Because of the different dipole moment directions in the Phe–Phe peptide compared to those in the Aβ polypeptide, an even smaller monomer, a single Phe amino acid, was studied. As illustrated in Figure 3a, the dipole moment in the Phe amino acid is pointing toward its aromatic ring, perpendicular to the fibril growth (similar to that of the protein). The strength of the dipole moment is much smaller than that of the full protein. In addition, to reduce sample-to-sample variations, the effect of different magnetization orientations within a single droplet was studied.

To achieve this, Amino acid solutions (l-Phe and d-Phe) were drop-cast onto a magnetic substrate with a wedge-shaped Au layer (0–10 nm thick). The Au layer limits spin penetration from the underlying ferromagnetic Co (Figure 3d), creating a spin-polarized (M) region on the thin Au side (<5 nm) and a nonspin-polarized (n-M) region on the thick Au side (>5 nm).^38^ Thus, two cases can be distinguished: an area with high spin interactions between chiral molecules and the cobalt layer and an area with low spin interactions due to a thicker Au cover layer, resulting in high spin scattering in the gold layer. For simplicity, further in text, those areas will be named as “magnetic” and “nonmagnetic” respectively. Due to the external magnet used, the assembly is done under a uniform weak magnetic field. However, one side of the sample is spin polarized (the thin Au side) and the other is not spin polarized (the opposite, thick Au side). This method also helps us differentiate between spin effects and magnetic field effects.

ThT fluorescence was used to evaluate the number of amyloid-like fibrils in both areas (magnetic (M) and nonmagnetic (n-M)) for each magnetization direction (Figure 3e). The left-handed amino acid displays higher fluorescence intensity on the South-magnetized substrate (M S = 161 ± 24) compared to the same substrate’s nonmagnetic area (n-M S = 107 ± 5). For assemblies on the North-magnetized substrate, comparable fluorescence was observed for the magnetic and nonmagnetic areas (M N = 139 ± 15, n-M N = 127 ± 17). Results for the right-handed amino acid yield the opposite effect: higher fluorescence for assembly on the North-magnetized substrate area (M N = 166 ± 17) compared to the same substrate’s nonmagnetic area (n-M N = 110 ± 6). On the South-magnetized substrate, there was no significant difference between the magnetic and nonmagnetic areas (M S = 111 ± 4, n-M S = 124 ± 12). Due to small experimental differences between the monomer solutions (i.e., l- and d-forms), we do not directly compare fluorescence intensities between chiralities. Instead, comparisons are made only within each sample—between the spin-polarized and nonspin-polarized regions—and across different magnetization orientations. Comparable fluorescence intensities are observed between all nonmagnetic areas, indicating the effects are not due to the external magnetic field.

Overall, the South-magnetized substrate encourages the assembly of the left-handed amino acid, while the North-magnetized substrate encourages the assembly of the right-handed amino acid, displaying a reversal of the spin effect due to the reversal of the chirality. While the two enantiomers have the same electric dipole, their spin polarization is opposite. The errors in fluorescence values are due to the fact that all the data were taken from the same drop, thus influencing the statistics. Moreover, in comparison to the nonmagnetic substrate area, the magnetic substrate area promotes the self-assembly process rather than hindering it.

### Structural Analysis of the Fibrils

ATR–FTIR experiments confirmed the fibril structures. The analysis was focused on the Amide I (1700–1600 cm^–1^) and Amide II (1600–1500 cm^–1^) regions. Different spectra were observed due to the difference in the monomers used. The recurring peaks at 1630 cm^–1^ (Aβ), 1685 cm^–1^ (Phe–Phe), and 1640 cm^–1^ (Phe) (see Figure 4) fit well the known signatures for β-sheets.^39,40^ The Phe and Phe–Phe spectra also show an additional peak at 1605 cm^–1^, indicative of the C–C ring.^41^ The Amide II range shows varying peaks (1580–1530 cm^–1^), corroborating the cross-β structure.

**Figure 4:**
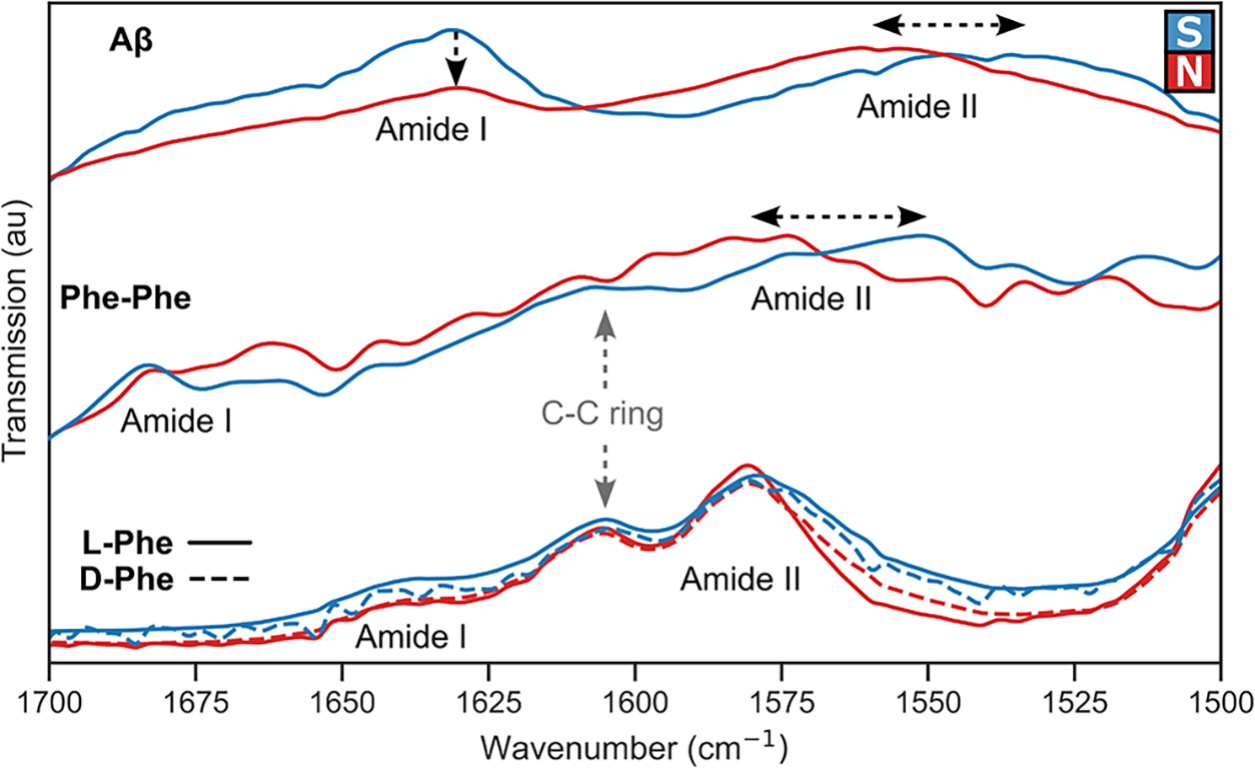
ATR–FTIR spectra of Aβ polypeptide (top), Phe–Phe dipeptide (middle), and l-Phe (solid line) and d-Phe (dashed line) amino acids (bottom) formed on magnetic surfaces magnetized in either the North (red) or South (blue) direction. The spectra show characteristic Amide I (1640–1630 cm^–1^) and Amide II (1600–1500 cm^–1^) peaks, indicating a cross-β structure, characteristic of amyloid-like fibrils. In Aβ, a decrease in the Amide I peak and a 30 cm^–1^ wavenumber shift toward higher frequencies are observed for the South to North magnetization directions. In Phe–Phe, only a shift is observed, with no decrease in transmission. In Phe, the spectra remain unchanged, indicating no structural differences between North and South magnetization directions, showing that instead the dynamics are dominant in the single monomer process. The Phe and Phe–Phe spectra also show an additional peak at 1605 cm^–1^, indicative of the C–C ring.

When comparing the spectra of Aβ prepared on the North or South magnetization orientation, two main differences are observed: a decrease in the intensity of the Amide I peak compared to the Amide II peak and a significant 30 wavenumber shift in Amide II peaks. The spectral changes slowly disappear in smaller monomers, Phe–Phe and Phe. In the Phe–Phe spectra, only the Amide II shift is observed, with no decrease. The Phe spectra remain unchanged, indicating no structural differences between North and South magnetization directions. The spectral changes repeated on different substrates and are presented in the Supporting Information.

## Discussion

The changes in amyloid-like self-assembly observed here can be divided into two types: morphological (amount and length) and structural. South magnetization consistently favored fibril formation (more fibrils, Figures 2c and 3e, and longer fibrils, Figure 3b,c) even for different L-chiral monomers. This can be interpreted as changes in the dynamics of the self-assembly process. The peptide assembly involves several successive stages of nucleation, elongation, and potentially hierarchical aggregation. These stages are governed by distinct physical constraints, discussed separately below.

During nucleation, previous studies have shown that the adsorption rates of chiral molecules on perpendicularly magnetized substrates are chirality-dependent. As the chiral molecule approaches the substrate surface, it undergoes charge reorganization, and therefore, spin polarization occurs due to the CISS effect. Thus, the spin-polarized substrate and molecule have either parallel (triplet-like) or antiparallel (singlet-like) spins, where the antiparallel case is energetically favored.^24,42^ This leads to a faster nucleation on the South magnetization orientation, as illustrated in Figure 5a. Since the experiments were done while drying the sample, the samples’ nucleation rates were not very sensitive to different monomer concentrations (see Figure S13 for more information). The changes in nucleation rates are also supported by observing the dynamics of Phe amino acid assembly using fluorescence microscopy, as seen in the Supporting Information Figure S14.

**Figure 5:**
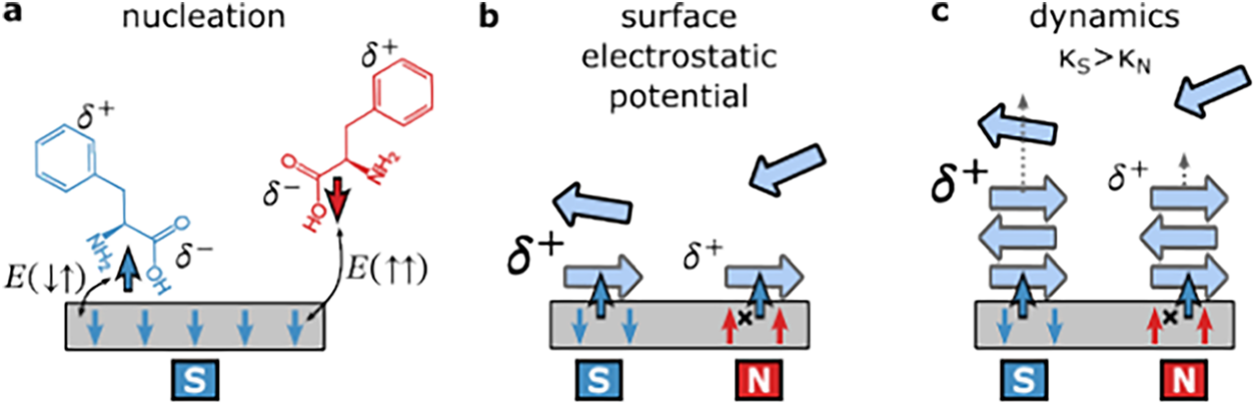
Proposed mechanism for spin-selective control of the amyloid assembly. (a) During the adsorption of l- or d-phe (blue/red) monomers onto a South-magnetized (blue) magnetic substrate, transient spin polarization occurs. The l-enantiomer forms an energetically favorable singlet-like (antiparallel spin) configuration with the spin-polarized substrate and is thus adsorbed more efficiently, initiating faster nucleation. The effect is reversed with chirality (d-enantiomer will prefer the North orientation). (b) After the nucleation forms, the South-magnetized interface (blue) results in a higher electrostatic potential compared to the North-magnetized (red) side, enhancing charge-mediated interactions between the first layer and the new monomers added to it. (c) Elevated surface potential leads to an increased fibril elongation rate (κ_*S*_ > κ_*N*_), amplifying the spin-selective effect throughout the fibril growth process.

In contrast, the elongation stage is governed by the thermodynamic recognition between monomers and growing fibril ends. Here, several studies have shown spin-dependent variations in the surface electrostatic potential of chiral molecules’ monolayer on a magnetic substrate^38,43,44^ (Figure 5b). These can be attributed either to conventional CISS-induced spin filtering^45,46^ or to magnetochiral charge-trapping effects at chiral/ferromagnetic interfaces.^47,48^ The former mechanism relies on spin exchange between transiently polarized electrons, while the latter involves modifications of the effective interfacial potential landscape without requiring spin-polarized carriers. Both will result in a change in interaction strength between monomers and therefore affect the elongation dynamics, as illustrated in Figure 5c.

These mechanisms would be especially prominent in a highly stereoselective system such as the antiparallel β-sheet structure. While α helix oligopeptides are a go-to model for the CISS effect due to their well-defined chiral structure and correlation with spin polarization,^13,49^ the spin-selective properties of β-sheet assemblies were explored in synthetic systems,^50,51^ but biologically relevant β-sheet assemblies remain less explored. In such systems, even subtle disruptions of molecular recognition can significantly affect the elongation rate constants.

Supporting this are the ATR–FTIR spectra. The spectral shifts between different magnetization orientations indicate structural differences between oligomers and mature fibrils.^40,52^ Both species are likely present in the sample, with their relative proportions varying. Notably, despite the fact that the South magnetization orientation exhibits higher fluorescence—suggesting increased amyloid content, its ATR–FTIR signature indicates a higher proportion of oligomers relative to mature fibrils.

Given our experimental system’s complexity and nonequilibrium nature, it is not currently possible to distinguish between these mechanisms. Future experiments incorporating time-resolved measurements, in-plane magnetization geometries, or under IR radiation, depleting surface traps that affect the nucleation stage may help elucidate the dominant microscopic mechanisms.

The single Phe amino acid monomers (Figure 3e) conveyed two important references. First, switching chirality alters the spin polarization, while the dipole remains unchanged, supporting the role of chirality in spin interactions. Although there are still open questions about the mechanism, this strongly supports a CISS-mediated origin for the observed phenomena. Second, distinct effects were observed on the same surface in different areas (magnetic and nonmagnetic), depending on the presence or absence of spin injection, indicating a localized influence of spin-polarized currents on the system and ruling out the magnetic field effect per se.

In addition, the different monomers explored in Figure 3 (A-β, Phe–Phe, and Phe) differ in their dipole moment strength and direction. Thus, the role of the monomer’s dipole moment was explored, showing that different dipole directions yielded different morphology on the magnetized substrate: shorter fibrils in Figure 3b,c versus changes in dynamics in Figures 2c and 3e. Specifically, the Phe–Phe case is interesting, where the monomer dipole moment is in the growth direction. In this case, a significant shortening of the fibrils was observed for the unfavored magnetization direction (Figure 3b,c). Studying smaller peptides such as Phe and Phe–Phe, which are more sensitive to surface effects, helps isolate surface-driven mechanisms. However, the observed behaviors suggest a more complex origin, involving both the dipole orientation and molecular structure.

Spin is inherently challenging to define and measure in biological systems since there is no clear axis in these systems, which has historically limited its investigation. However, by conducting the self-assembly process on a magnetic substrate and connecting the spin to the chiral structure of the fibril, it becomes possible to define a specific spin direction. Moreover, spin-exchange interactions, while very short-ranged, are extremely strong. Here, spin-exchange interactions are introduced to the system by a spin-polarized thin ferromagnetic layer and the chirality of the monomers, resulting in significant effects by using low magnetic fields.

Functionalized substrates offer a unique platform for controlling amyloid self-assembly, as aggregation is highly sensitive to surface properties.^34^ As delivery to target sites remains a major challenge, an alternative approach is integrating such surfaces into blood dialysis to treat dialysis-related amyloidosis. Further research is needed regarding the generality of the effect. Its strength will likely depend on the molecule’s electric dipole. While the present work demonstrates directional control over fibril dynamics via surface magnetization orientation, future systems, such as assembly in flow systems on magnetized nanoparticles or high-surface-area filters, could enable continuous, tunable modulation of amyloid formation, offering more precise control over both kinetics and selectivity.

## Conclusions

In spite of its key role in amyloid formation, the role of spin interactions in this process was never explored. Our study reveals that transient spin polarization, driven by the CISS effect, plays a significant role in amyloid-like fibril formation. It is important to note that electronic spin effects are rather unexpected in biological systems, which are large and scattered. The average length of a single fibril is 200 nm, and the aggregation is 1–100 μm in size. Both are significantly longer than the expected length for spin effects at these conditions, which is usually in the few Å range.^53^ This is likely because we are changing the dynamic behavior through dipole interactions in the self-assembly process.

Using different spin-polarized interfaces, we observe a preferential magnetization orientation for fibril formation, resulting in up to twice as many fibrils being formed and significantly longer fibrils in the favored magnetization direction. So by tailoring the spin-polarized interface, we were able to control the amyloid assembly process. The monomer chirality and dipole moment significantly alter the favored magnetization orientation. In addition, structural changes, such as shifts between oligomers to mature fibrils between samples, were observed, supporting the unexpected role of spin interactions in protein structural dynamics. Despite the expected short-range nature of the spin interactions, the effects persist at much larger scales. These findings not only provide insights into an additional mechanism behind amyloid formation but also open possibilities for using magnetic particles in therapeutic strategies for amyloid-related disorders. Specifically, using magnetic nanoparticles to selectively modulate amyloid formation in the treatment of neurodegenerative diseases and integrating spin-selective interfaces in dialysis systems to mitigate dialysis-related amyloidosis.

## Experimental Section

### Materials

The l-phe, d-phe, and diphenylalanine were purchased from Sigma. The diphenylalanine analog Ac-Phe-Phe-NH_2_ and amyloid β-polypeptide (HFIP-treated) were purchased from Bachem.

### Sample Preparation

Allocates of the monomerized A-β (1–42) polypeptide were prepared by dissolving 1 mg A-β (1–42) in 1 mL of high-grade 1,1,1,3,3,3-hexafluoroisopropanol (HFIP, Sigma) by 20s sonication on ice then by constant shaking at 150 rpm at 37 C for 90 min. The samples were then aliquoted into 200 μg stocks, and the solvent was left to evaporate before storage at −20 C until use.

Fresh stocks of A-β (1–42) polypeptide solution were prepared by dissolving 200 μg A-β (1–42) in 42.9 μL dimethyl sulfoxide (DMSO, Sigma) by 60s sonication. The solution was diluted in 1066 μL of PBS (pH 7.4) to achieve a final concentration of 40 μM. The solution was dyed with Thioflavin T (ThT, Sigma) at a concentration of 40 μM. 5 μL drops of the final solution were drop-casted onto magnetic substrates, magnetized by placement on a permanent magnet oriented in the South or North direction. The droplet was then allowed to dry and self-assemble into fibrils at 37 C. Two samples were made for each magnetization orientation to reduce variability.

Fresh stock solutions of the diphenylalanine were prepared by dissolving the lyophilized peptide in HFIP at a concentration of 100 mg/mL. The peptide stock solution was diluted to a final concentration of 2 mg/mL.^39^ The solution was dyed with ThT at a concentration of 10 μM.

Fresh stock solutions of all other peptides and amino acids were prepared by dissolving the lyophilized peptides in ddH_2_O at 90 C for 3 h and diluting to a final concentration of 0.1 mg/mL similar to.^6^ The solution was dyed with ThT at a concentration of 10 μM.

To avoid any preaggregation, fresh stock solutions were prepared for each experiment. The final solutions were drop-casted (5 μL drops) onto magnetic substrates, magnetized by placement on a permanent magnet (75 mT) oriented to South or North directions. The droplet was then allowed to dry and self-assemble into fibrils at room temperature. Droplets from the same stock solution were drop-cast onto two substrates (for each magnetization: North, South) to reduce variability.

### Magnetic Substrates Growth

Molecular beam epitaxy (MBE) grown epitaxial thin film magnetic samples with perpendicular anisotropy (Al_2_O_3_ (0001)/ Pt­(50Å)/Au­(200Å)/Co­(13Å)/Au­(50Å)) were used for the experiments. Magnetization reorientation of the FM samples was performed by an external magnetic field of 75 mT in a perpendicular direction (South or North, respectively) at room temperature. The coercive field of the FM samples used was ∼25 or ∼12 mT (for the Au wedge samples). The FM samples’ easy axis was out of-plane (OOP), thus ensuring that the applied magnetic field would reorient the magnetization OOP, parallel or antiparallel to the surface normal.

Epitaxial Au wedge samples of the same cobalt thickness were also grown by MBE with a configuration: Al_2_O_3_(0001)/Pt­(50Å)/Au­(200Å)/Co­(13Å)/Au­(0–100Å). The magnetic (M) areas were measured at the 2–5 nm Au thickness area, while the nonmagnetic (n-M) areas were measured at 6–8 nm thickness of the Au layer. The small variations in Au thickness areas studied between different samples were due to slightly different locations of the solution droplet.

For the FTIR on Phe solutions and the experiments with the peptide analogue, Ti (2 nm)/Ni (80 nm)/Au (5 nm) film was grown using AJA ATC Polaris Series UHV sputtering system (base Pressure: 3 × 10^–9^ Torr) onto a Si/SiO wafer.

### Scanning Electron Microscopy

Scanning electron microscopy (SEM) images for the A-β polypeptide and Phe–Phe peptide samples were acquired using an Extra-High-Resolution Scanning Electron Microscope Magellan 400L (ThermoFisher, formerly FEI). The imaging was conducted at a current of 25 pA and an accelerating voltage of 5 kV, with a working distance of 4.1 mm and a magnification of 350X. Both secondary electron and backscattered electron imaging modes were used. For the Phe amino acid samples, imaging was performed using an Analytical High Resolution Scanning Electron Microscope Apreo 2S (ThermoFisher Scientific) at a current of 0.1 nA and an accelerating voltage of 2 kV, with a working distance of 4.6 mm and a magnification of 350X. Each measurement of intensity value shown is taken from 3 different locations on the substrate to reduce local variability.

### Transmission Electron Microscopy

Samples (10 μL drop) were placed on glow-discharged carbon-coated 300 mesh copper TEM grids (Ted Pella, Inc.). After blotting, the samples were either dried in air before observation or negatively stained with 2% aqueous solution of uranyl acetate for 2 min and air-dried. The samples were examined by a FEI Tecnai 12 G2 TWIN TEM operated at 120 kV. Images were recorded using a 4k × 4k FEI Eagle CCD camera.

Direct imaging of the samples in their native, aqueous environment was performed by using cryogenic transmission electron microscopy (cryo-TEM). In this method, a drop (2.5 μL) of the solution was deposited on a glow-discharged TEM grid (300 mesh Cu Lacey substrate, Ted Pella, Ltd.). Vitrobot Mark IV (FEI) was used to blot the excess liquid in a controlled environment and to vitrify the specimens by a rapid plunging into liquid ethane precooled with liquid nitrogen. The vitrified samples were examined at −179C using a FEI Tecnai 12 G2 TWIN TEM operated at 120 kV and equipped with a Gatan 626 cold stage. TIA (Tecnai Imaging and Analysis) software was used to record the images in low-dose mode on a 4K × 4K FEI Eagle CCD camera.

### Fourier Transform Infrared Spectroscopy

To assess conformational changes in the fibril structure between the different samples, ATR–FTIR spectra of the amyloid-like structures were recorded using a Thermo Scientific Nicolet iS50 FTIR spectrometer with an ATR accessory with a MCT detector, using a resolution of 4 cm^–1^ and averaging 32 scans per spectrum over the range of 4000–600 cm^–1^.

### Thioflavin T Binding Assay

ThT was imaged using the FV-1200 confocal microscope with a 10×/0.45 objective (Olympus, Japan), excited at 405 nm and collected at 450–550 nm. A bright-field image was collected as well. Z-stack images were obtained at 2 μm distance. Time-lapse was also obtained for monitoring the assembly of the fibrils. Each measurement of intensity value shown is taken from 3 different locations on the substrate, to reduce local variability.

## Supplementary Material



## Data Availability

All data are available in the main text or the Supporting Information. Full confocal image files are available at 10.5281/zenodo.14925254

## Notes

Supporting text and Figures S1–S11, including sample images, characterization of magnetic substrates, spin selectivity calculations, additional TEM and SEM analysis, confocal images, FTIR analysis protocol, and additional experiments with charge-termini peptide (PDF)

## References

[1] Knowles T. P. J., Vendruscolo M., Dobson C. M. (2014). The amyloid state and its association
with protein misfolding diseases. Nat. Rev.
Mol. Cell Biol..

[2] Wei G., Su Z., Reynolds N. P., Arosio P., Hamley I. W., Gazit E., Mezzenga R. (2017). Self-assembling peptide and protein amyloids: from
structure to tailored function in nanotechnology. Chem. Soc. Rev..

[3] Reches M., Gazit E. (2003). Casting metal nanowires within discrete self-assembled peptide nanotubes. Science.

[4] Soto C., Sigurdsson E. M., Morelli L., Asok Kumar R., Castaño E. M., Frangione B. (1998). *β*-sheet breaker
peptides inhibit fibrillogenesis in a rat brain model of amyloidosis:
implications for Alzheimer’s therapy. Nat. Med..

[5] Tjernberg L.
O., Näslund J., Lindqvist F., Johansson J., Karlström A. R., Thyberg J., Terenius L., Nordstedt C. (1996). Arrest of-Amyloid
Fibril Formation by a Pentapeptide Ligand (*). J. Biol. Chem..

[6] Adler-Abramovich L., Vaks L., Carny O., Trudler D., Magno A., Caflisch A., Frenkel D., Gazit E. (2012). Phenylalanine assembly
into toxic fibrils suggests amyloid etiology in phenylketonuria. Nat. Chem. Biol..

[7] Zaguri D., Zimmermann M. R., Meisl G., Levin A., Rencus-Lazar S., Knowles T. P., Gazit E. (2021). Kinetic and thermodynamic driving
factors in the assembly of phenylalanine-based modules. ACS Nano.

[8] Pandey G., Saikia J., Sasidharan S., Joshi D. C., Thota S., Nemade H. B., Chaudhary N., Ramakrishnan V. (2017). Modulation
of peptide based nano-assemblies with electric and magnetic fields. Sci. Rep..

[9] Wang M., Du L., Wu X., Xiong S., Chu P. K. (2011). Charged diphenylalanine
nanotubes and controlled hierarchical self-assembly. ACS Nano.

[10] A
Hill R., Sedman V. L., Allen S., Williams P., Paoli M., Adler-Abramovich L., Gazit E., Eaves L., Tendler S. J. (2007). Alignment
of aromatic peptide tubes in strong magnetic fields. Adv. Mater..

[11] Nguyen V., Zhu R., Jenkins K., Yang R. (2016). Self-assembly of diphenylalanine
peptide with controlled polarization for power generation. Nat. Commun..

[12] Su Y., Liu J., Yang D., Hu W., Jiang X., Wang Z. L., Yang R. (2023). Electric field-assisted
self-assembly of diphenylalanine peptides
for high-performance energy conversion. ACS
Mater. Lett..

[13] Göhler B., Hamelbeck V., Markus T., Kettner M., Hanne G., Vager Z., Naaman R., Zacharias H. (2011). Spin selectivity
in electron transmission through self-assembled monolayers of double-stranded
DNA. Science.

[14] Xie Z., Markus T. Z., Cohen S. R., Vager Z., Gutierrez R., Naaman R. (2011). Spin specific electron
conduction through DNA oligomers. Nano Lett..

[15] Mishra D., Markus T. Z., Naaman R., Kettner M., Göhler B., Zacharias H., Friedman N., Sheves M., Fontanesi C. (2013). Spin-dependent
electron transmission through bacteriorhodopsin embedded in purple
membrane. Proc. Natl. Acad. Sci. U.S.A..

[16] Niman C. M., Sukenik N., Dang T., Nwachukwu J., Thirumurthy M. A., Jones A. K., Naaman R., Santra K., Das T. K., Paltiel Y. (2023). Bacterial
extracellular
electron transfer components are spin selective. J. Chem. Phys..

[17] Varade V., Markus T., Vankayala K., Friedman N., Sheves M., Waldeck D. H., Naaman R. (2018). Bacteriorhodopsin
based non-magnetic
spin filters for biomolecular spintronics. Phys.
Chem. Chem. Phys..

[18] Gupta R., Chinnasamy H. V., Sahu D., Matheshwaran S., Sow C., Chandra Mondal P. (2023). Spin-dependent electrified protein
interfaces for probing the CISS effect. J. Chem.
Phys..

[19] Sang Y., Mishra S., Tassinari F., Karuppannan S. K., Carmieli R., Teo R. D., Migliore A., Beratan D. N., Gray H. B., Pecht I. (2021). Temperature dependence
of charge and spin transfer in azurin. J. Phys.
Chem. C.

[20] Al-Bustami H., Belsey S., Metzger T., Voignac D., Yochelis S., Shoseyov O., Paltiel Y. (2022). Spin-induced organization of cellulose
nanocrystals. Biomacromolecules.

[21] Aminadav G., Shoseyov O., Belsey S., Voignac D., Yochelis S., Levi-Kalisman Y., Yan B., Shoseyov O., Paltiel Y. (2024). Chiral Nematic
Cellulose Nanocrystal Films for Enhanced Charge Separation and Quantum-Confined
Stark Effect. ACS Nano.

[22] Tassinari F., Steidel J., Paltiel S., Fontanesi C., Lahav M., Paltiel Y., Naaman R. (2019). Enantioseparation
by
crystallization using magnetic substrates. Chem.
Sci..

[23] Naaman R., Paltiel Y., Waldeck D. H. (2019). Chiral
molecules and the electron
spin. Nat. Rev. Chem..

[24] Banerjee-Ghosh K., Ben Dor O., Tassinari F., Capua E., Yochelis S., Capua A., Yang S.-H., Parkin S. S., Sarkar S., Kronik L. (2018). Separation
of enantiomers by their enantiospecific
interaction with achiral magnetic substrates. Science.

[25] Lu Y., Bloom B., Qian S., Waldeck D. (2021). Enantiospecificity
of cysteine adsorption on a ferromagnetic surface: Is it kinetically
or thermodynamically controlled?. J. Phys. Chem.
Lett..

[26] Safari M. R., Matthes F., Caciuc V., Atodiresei N., Schneider C. M., Ernst K.-H., Bürgler D. E. (2024). Enantioselective
adsorption on magnetic surfaces. Adv. Mater..

[27] Banerjee-Ghosh K., Ghosh S., Mazal H., Riven I., Haran G., Naaman R. (2020). Long-range charge reorganization
as an allosteric control
signal in proteins. J. Am. Chem. Soc..

[28] Kumar A., Capua E., Kesharwani M. K., Martin J. M., Sitbon E., Waldeck D. H., Naaman R. (2017). Chirality-induced
spin polarization
places symmetry constraints on biomolecular interactions. Proc. Natl. Acad. Sci. U.S.A..

[29] Wei J., Bloom B. P., Dunlap-Shohl W. A., Clever C. B., Rivas J. E., Waldeck D. H. (2023). Examining the effects
of homochirality for electron
transfer in protein assemblies. J. Phys. Chem.
B.

[30] Kettner M., Gohler B., Zacharias H., Mishra D., Kiran V., Naaman R., Fontanesi C., Waldeck D. H., Sek S., Pawłowski J., Juhaniewicz J. (2015). Spin filtering in electron transport
through chiral oligopeptides. J. Phys. Chem.
C.

[31] Tassinari F., Amsallem D., Bloom B. P., Lu Y., Bedi A., Waldeck D. H., Gidron O., Naaman R. (2020). Spin-dependent enantioselective
electropolymerization. J. Phys. Chem. C.

[32] Bhowmick D. K., Das T. K., Santra K., Mondal A. K., Tassinari F., Schwarz R., Diesendruck C. E., Naaman R. (2022). Spin-induced asymmetry
reaction–The formation of asymmetric carbon by electropolymerization. Sci. Adv..

[33] Stovbun S. V., Zanin A. M., Skoblin A. A., Zlenko D. V. (2020). The weak magnetic
field inhibits the supramolecular self-ordering of chiral molecules. Sci. Rep..

[34] Grigolato F., Arosio P. (2021). The role of surfaces
on amyloid formation. Biophys. Chem..

[35] Brack A., Spach G. (1980). *β*-structures of polypeptides with L-and D-residues:
Part III. Experimental Evidences for Enrichment in Enantiomer. J. Mol. Evol..

[36] Muscat S., Stojceski F., Danani A. (2020). Elucidating the effect of static
electric field on amyloid beta 1–42 supramolecular assembly. J. Mol. Graphics Modell..

[37] Lührs T., Ritter C., Adrian M., Riek-Loher D., Bohrmann B., Döbeli H., Schubert D., Riek R. (2005). 3D structure
of Alzheimer’s amyloid-*β* (1–42)
fibrils. Proc. Natl. Acad. Sci. U.S.A..

[38] Ghosh S., Mishra S., Avigad E., Bloom B. P., Baczewski L., Yochelis S., Paltiel Y., Naaman R., Waldeck D. H. (2020). Effect
of chiral molecules on the electron’s spin wavefunction at
interfaces. J. Phys. Chem. Lett..

[39] Reches M., Gazit E. (2005). Self-assembly of peptide
nanotubes and amyloid-like structures by
charged-termini-capped diphenylalanine peptide analogues. Isr. J. Chem..

[40] Sarroukh R., Goormaghtigh E., Ruysschaert J.-M., Raussens V. (2013). ATR-FTIR: A “rejuvenated”
tool to investigate amyloid proteins. Biochim.
Biophys. Acta, Biomembr..

[41] Barth A. (2000). The infrared
absorption of amino acid side chains. Prog.
Biophys. Mol. Biol..

[42] Ziv A., Saha A., Alpern H., Sukenik N., Baczewski L. T., Yochelis S., Reches M., Paltiel Y. (2019). AFM-based spin-exchange
microscopy using chiral molecules. Adv. Mater..

[43] Abendroth J. M., Cheung K. M., Stemer D. M., El Hadri M. S., Zhao C., Fullerton E. E., Weiss P. S. (2019). Spin-dependent ionization
of chiral
molecular films. J. Am. Chem. Soc..

[44] Theiler P. M., Ritz C., Hofmann R., Stemmer A. (2023). Detection of a chirality-induced
spin selective quantum capacitance in *α*-helical
peptides. Nano Lett..

[45] Kapon Y., Saha A., Duanis-Assaf T., Stuyver T., Ziv A., Metzger T., Yochelis S., Shaik S., Naaman R., Reches M., Paltiel Y. (2021). Evidence for
new enantiospecific
interaction force in chiral biomolecules. Chem.

[46] Geyer M., Gutierrez R., Mujica V., Silva J., Dianat A., Cuniberti G. (2022). The contribution
of intermolecular spin interactions
to the London dispersion forces between chiral molecules. J. Chem. Phys..

[47] Tirion S. H., van Wees B. J. (2024). Mechanism for electrostatically
generated magnetoresistance
in chiral systems without spin-dependent transport. ACS Nano.

[48] Zhao Y., Zhang K., Xiao J., Sun K., Yan B. (2025). Magnetochiral
charge pumping due to charge trapping and skin effect in chirality-induced
spin selectivity. Nat. Commun..

[49] Eckshtain-Levi M., Capua E., Refaely-Abramson S., Sarkar S., Gavrilov Y., Mathew S. P., Paltiel Y., Levy Y., Kronik L., Naaman R. (2016). Cold denaturation induces
inversion of dipole and spin
transfer in chiral peptide monolayers. Nat.
Commun..

[50] Firouzeh S., Illescas-Lopez S., Hossain M. A., Cuerva J. M., Alvarez
de Cienfuegos L., Pramanik S. (2023). Chirality-induced spin selectivity
in supramolecular chirally functionalized graphene. ACS Nano.

[51] Rahman M. W., Mañas-Torres M. C., Firouzeh S., Illescas-Lopez S., Cuerva J. M., Lopez-Lopez M. T., de Cienfuegos L. Á., Pramanik S. (2022). Chirality-induced spin selectivity in heterochiral
short-peptide–carbon-nanotube hybrid networks: Role of supramolecular
chirality. ACS Nano.

[52] Sarroukh R., Cerf E., Derclaye S., Dufrêne Y. F., Goormaghtigh E., Ruysschaert J.-M., Raussens V. (2011). Transformation of amyloid *β* (1–40)
oligomers into fibrils is characterized
by a major change in secondary structure. Cell.
Mol. Life Sci..

[53] Metzger T. S., Mishra S., Bloom B. P., Goren N., Neubauer A., Shmul G., Wei J., Yochelis S., Tassinari F., Fontanesi C. (2020). The electron spin as
a chiral reagent. Angew. Chem..

