# Supplementary material for: Controlling Amyloid Assembly Dynamics Using Spin Interfaces
Source: ACS Nano. 2025 Jul 28;19(31):28326–34. doi: 10.1021/acsnano.5c06285 (PMC12356117; doi:10.1021/acsnano.5c06285)
Supplement: Supplementary file 1 [file nn5c06285_si_001.pdf]

Controlling Amyloid Assembly Dynamics Using Spin Interfaces

Yael Kapon,[†,#] Dror Merhav,[†,#] Gal Finkelstein-Zuta,[‡] Omer Blumen,[¶] Naomi Melamed-Book,[§] Yael Levi-Kalisman,[‖] Ilya Torchinsky,[‖] Shira Yochelis,[†] Daniel Sharon,[¶] Lech Tomasz Baczewski,[⊥] Ehud Gazit,[‡] and Yossi Paltiel[*,†]

†*Institute of Applied Physics, The Hebrew University, Jerusalem 9190401, Israel*

‡*The Shmunis School of Biomedicine and Cancer Research, George S. Wise Faculty of Life Sciences, Tel Aviv University, Tel Aviv 6997801, Israel*

¶*Institute of Chemistry, Hebrew University of Jerusalem, Jerusalem 91904, Israel*

§*Bio-Imaging Unit, The Alexander Silberman Institute of Life Science, The Hebrew University, Jerusalem 9190401, Israel*

‖*The Harvey M. Krueger Family Center for Nanoscience and Nanotechnology, The Hebrew University, Jerusalem 9190401, Israel*

⊥*Institute of Physics, Polish Academy of Sciences, Warsaw 02668, Poland*

#*These authors contributed equally.*

E-mail: paltiel@mail.huji.ac.il

Supporting Information

Sample Images

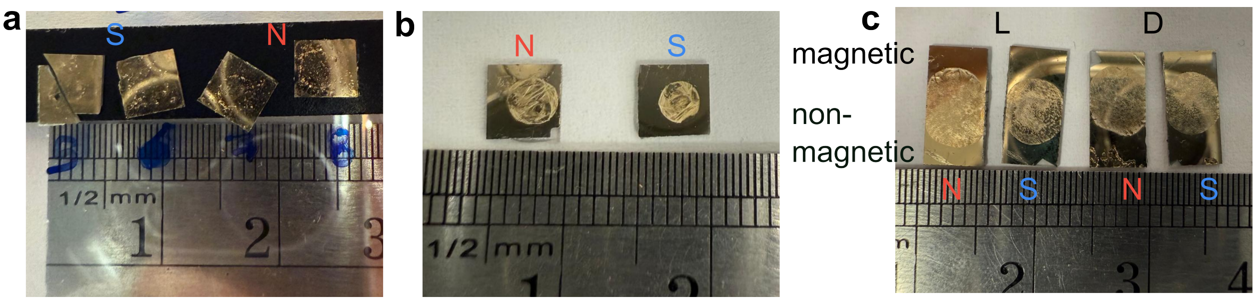

Figure S1: Optical images of the samples. **a** A-β polypeptide solution dried on an Al_2O_3 (0001)/Pt(50Å)/Au(200Å)/Co(13Å)/Au(50Å) substrate with perpendicular magnetic anisotropy (PMA) magnetized in the North or South direction. **b** Phe-Phe peptide solution dried on an Al_2O_3 (0001)/Pt(50Å)/Au(200Å)/Co(13Å)/Au(50Å) substrate magnetized in the North or South direction. **c** Phe amino acid solution dried on an Al_2O_3 (0001)/Pt(50Å)/Au(200Å)/Co(13Å)/Au(0-100Å) substrate, with a wedge-shaped Au capped layer, magnetized in the North or South direction. The sample's thick (thin) Au side is marked as non-magnetic (magnetic).

Characterization of Magnetic Substrates

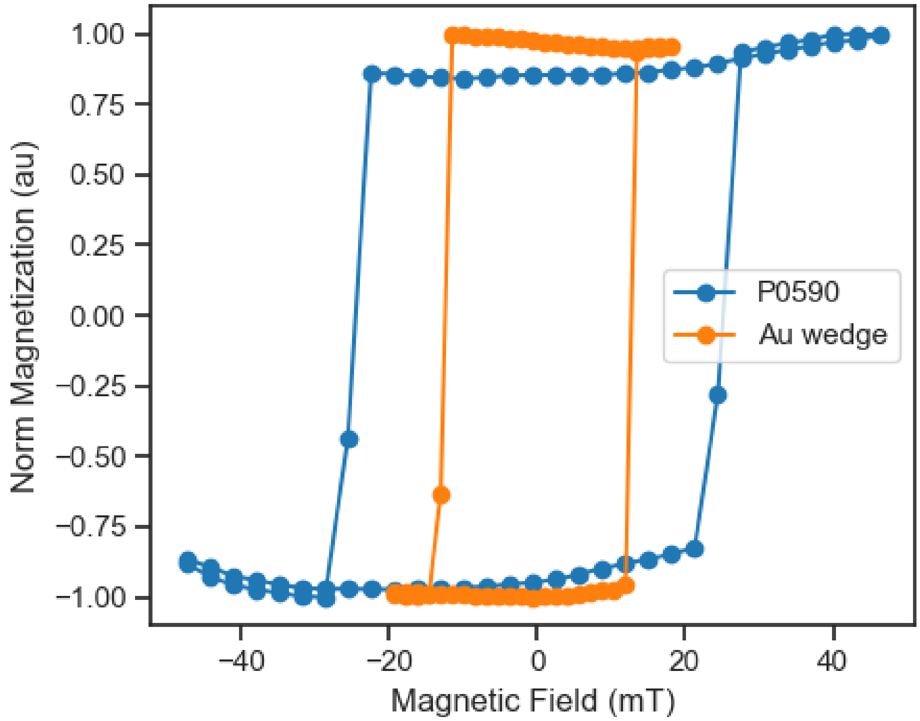

Figure S2: Magnetic hysteresis loops of Al_2O_3 (0001)/Pt(50Å)/Au(200Å)/Co(13Å)/Au(50Å) (P0590) (blue) and Au wedge (orange) substrate at Au thickness of 4 Åmeasured using MOKE microscope

The magnetic properties of the substrates were characterized using a magneto-optical Kerr effect (MOKE) microscope. MOKE measurements were performed using a commercial Evico Magnetics GmbH magneto-optical Kerr microscope, equipped with an electromagnet and a piezo controller for mechanical stabilization. Magnetic hysteresis loops were obtained by sweeping the applied magnetic field from negative to positive and back to negative. The measured hysteresis loops are shown in Figure S2, with the blue curve corresponding to the Al_2O_3 (0001)/Pt(50Å)/Au(200Å)/Co(13Å)/Au(50Å) (P0590) substrate and the orange curve representing the Au wedge substrate measured for Au thickness of 4 Å. The P0590 substrate exhibited a coercive field of 25 mT, while the wedge substrate had a lower coercive field of 12 mT.

Spin Selectivity

To quantify spin selectivity (relative difference in fluorescence intensity), we calculate the relative difference in fluorescence intensity between the South and North magnetized substrates, normalized by their sum. The spin selectivity (relative difference in fluorescence intensity) (P) is given by:

$$P = \frac{I_S - I_N}{I_S + I_N} \times 100$$

To calculate the uncertainty in spin selectivity (P), we use the error propagation formula. For a function $P = f(I_S, I_N)$, the propagated uncertainty ΔP is:

$$\Delta P = \sqrt{\left(\frac{\partial P}{\partial I_S}\Delta I_S\right)^2 + \left(\frac{\partial P}{\partial I_N}\Delta I_N\right)^2}$$

Substituting these into the uncertainty formula gives:

$$\Delta P = \sqrt{\left(\frac{2I_N}{(I_S + I_N)^2}\Delta I_S \times 100\right)^2 + \left(\frac{-2I_S}{(I_S + I_N)^2}\Delta I_N \times 100\right)^2}$$

We get a spin selectivity (relative difference in fluorescence intensity) of: P=27±10% for the polypeptide, P=5±9% for the Phe-Phe peptide, P = 7 ± 9% for L-Phe and P = 19 ± 6% for D-Phe.

TEM analysis

A-β in Solution VS. on a Substrate

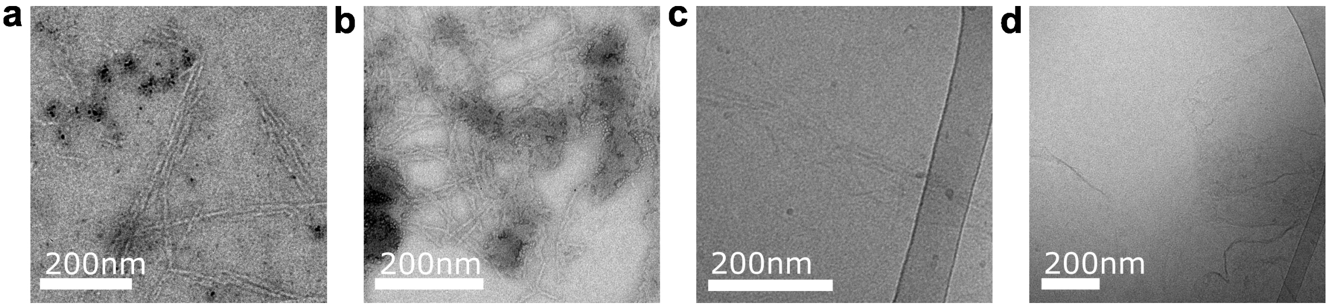

Figure S3: TEM images of A-β polypeptide. Negatively stained images showing the fibrillar aggregates both fresh **a**, and after 3 days in the solution **b**. Cryo-TEM images showing fibrillar aggregates **c** and thin sheets **d**.

The TEM imaging of negatively stained solution of A-β in PBS revealed that the A-β polypeptide self-assembled into fibrillar structures (Figure S3a). Similar structures were observed when the solution was left to age for three days at room temperature (Figure S3b). Cryo-TEM was employed to study the aggregation process in solution without drying the sample on a substrate. A vitrified sample of A-β solution in PBS showed both fibrillar structures, similar to the structures formed on the substrates (Figure S3c) as well as some thin wide sheets (Figure S3d).

TEM of Phe-Phe Peptide

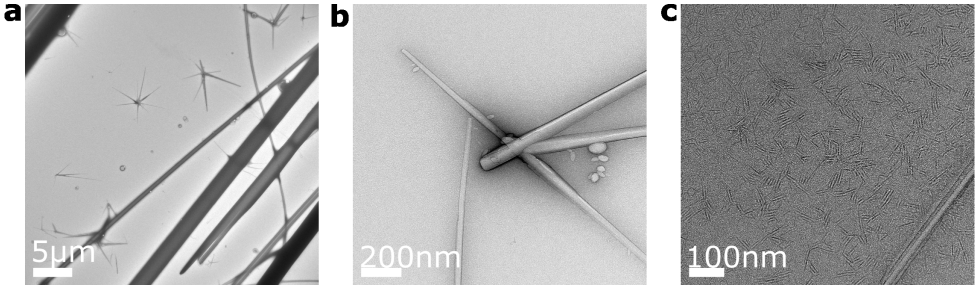

Figure S4: TEM images of negatively stained sample of PhePhe peptide at different magnifications showing structures that range in size, from large structures a few tens of microns long and a few microns in diameter (**a**) to a few hundreds nanometers long and a few tens of nm in diameter (**b**). Very short, rod-like structures (about 20 nm long and 2 nm in diameter) were also observed in many areas around the large structures (**c**).

SEM Analysis

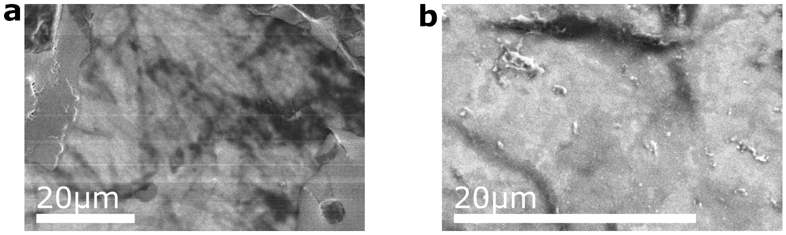

Figure S5: Secondary electron microscopy (SEM) images of A-β polypeptide aggregates. **a** At 800× magnification, elongated fibrils can be observed bridging two larger aggregates. **b** At 2000× magnification, darker fibrils are visible, though their fine details are not resolved, along with smaller, brighter aggregates indicating variations in surface topography. Scale bar: 20 μm.

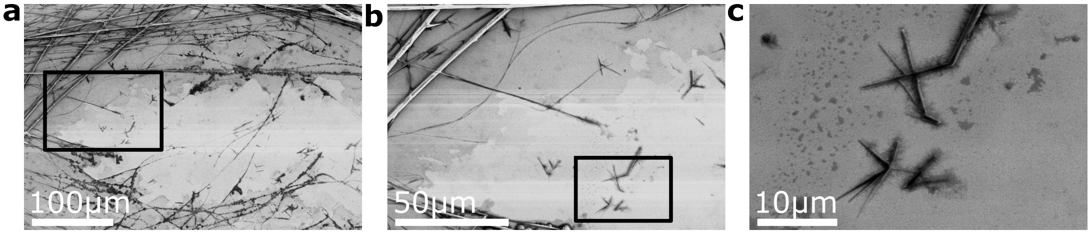

Figure S6: Secondary electron microscopy (SEM) images of Phe-Phe peptide fibrils. **a** At 350× magnification, a variety of fibrils are observed, including long fibrillar structures (>500 μm) and shorter ones (∼10 μm), some of which are coated with aggregates. The black rectangle indicates the region enlarged in (b). **b** At 1000× magnification, a closer view of the shorter fibrillar structures is shown. The black rectangle marks the area further enlarged in (c). **c** At 3500× magnification, the shorter fibrillar structures appear coated with much smaller fibrillar aggregates.

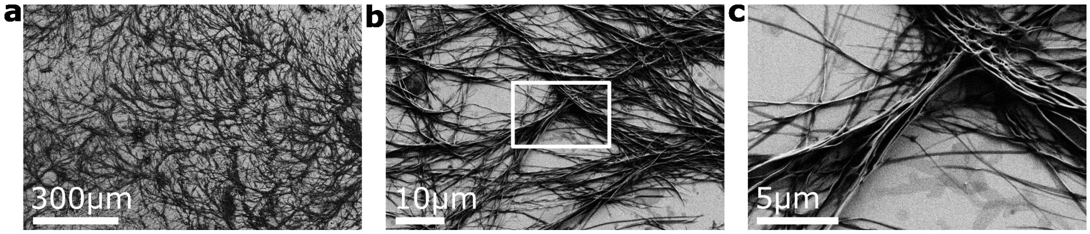

Figure S7: Secondary electron microscopy (SEM) images of L-Phe amino acid fibrils, acquired at 0.1 nA and 2 kV. **a** At 350× magnification, fibrillar structures are observed forming bundled arrangements. **b** At 6000× magnification, a closer view reveals fibrillar structures with diameters of approximately 500 nm. The white rectangle marks the region enlarged in (c). **c** At 20,000× magnification, a detailed view of the fibrillar structures is shown.

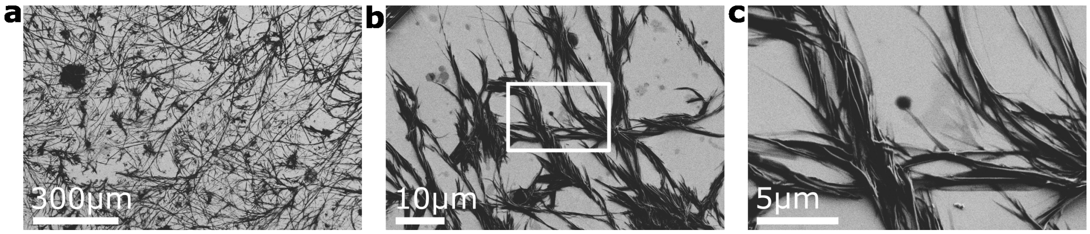

Figure S8: Secondary electron microscopy (SEM) images of D-Phe amino acid fibrils, acquired at 0.1 nA and 2 kV. **a** At 350× magnification, fibrillar structures are observed forming bundled arrangements. **b** At 6000× magnification, a closer view reveals fibrillar structures with diameters of approximately 500 nm. The white rectangle marks the region enlarged in (c). **c** At 20,000× magnification, a detailed view of the fibrillar structures is shown. The fibrils seem shorter han the L-chirality fibrils.

Confocal Images Analysis

A-β polypeptide and Phe-Phe Peptide

Fluorescence intensity and its error are calculated by acquiring three confocal fluorescence images for each of the two samples. For each image, a Z-projection is performed using the ImageJ program to obtain an average-intensity image, from which the mean fluorescence intensity across the entire image is computed. These mean intensities are then averaged over all three images to determine the final average fluorescence signal (in arbitrary units, au) for each sample. The error is represented by the standard error of the mean (SEM) calculated from the fluorescence intensities of the different images.

Phe Amino Acid

Fluorescence intensity and its error are calculated by acquiring fluorescence images from the magnetic or non-magnetic area of the sample. For each image, a Z-projection is performed using the ImageJ program to obtain an average intensity image, and a region of interest (ROI) is selected to ensure sufficient distance from the edge of the drop, avoiding the effects of the coffee ring. The ROI is then divided into four parts, and the mean intensity is calculated for each. These mean intensities are averaged to determine the final average fluorescence signal (in arbitrary units, au) for each sample. The error is represented by the standard error of the mean (SEM) calculated from the fluorescence intensities of the four areas.

Image Files

Full image files are available at https://doi.org/10.5281/zenodo.14925254

Baseline ThT Fluorescence

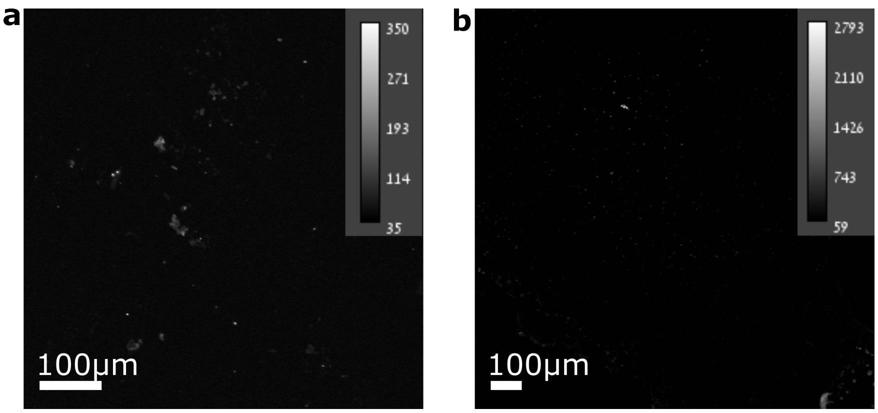

Figure S9: Z-projection images of ThT fluorescence intensity obtained using ImageJ. **a** 40 μM ThT in PBS, representing the baseline fluorescence intensity for A-β polypeptide samples, with an average intensity of 48. **b** 10 μM ThT in water, representing the baseline fluorescence intensity for Phe-Phe peptide and L-Phe amino acid samples, with an average intensity of 82.

ThT Fluorescence Spectrum

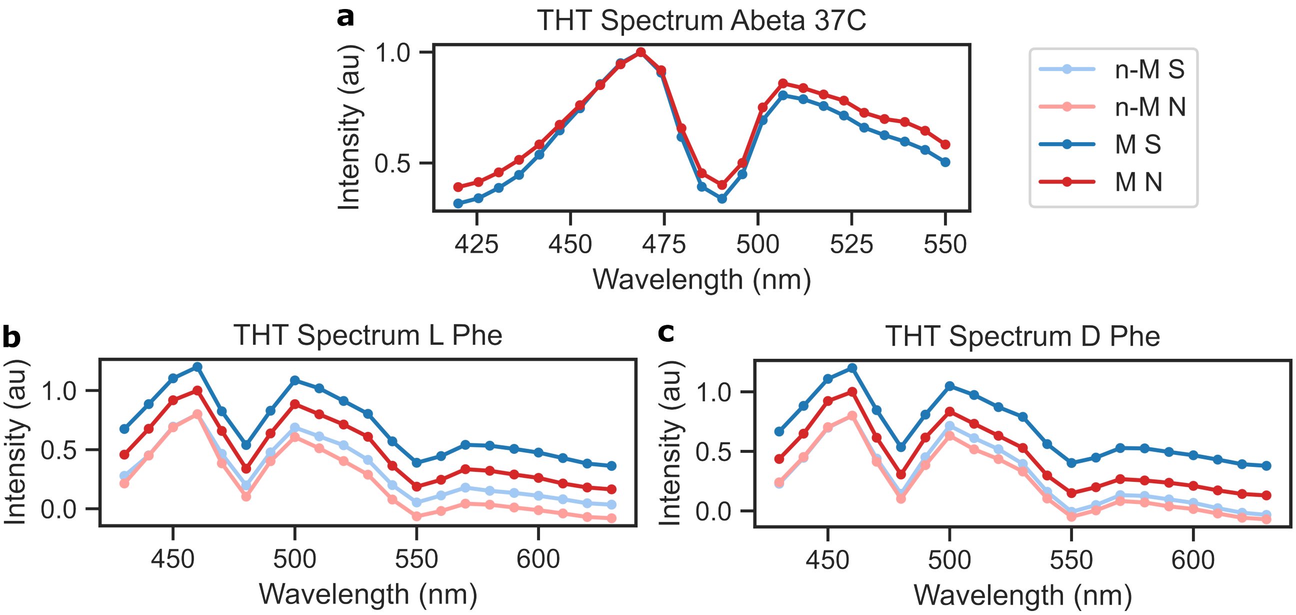

Figure S10: Fluorescence spectra of ThT for the samples. The spectra remain unchanged between different samples and magnetization orientations. The South-oriented spectra are shown in blue, North-oriented spectra in red, and nonmagnetic sides in light blue/red. **a** Fluorescence spectra of the A-β sample. **b** Fluorescence spectra of the L-Phe sample. **c** Fluorescence spectra of the D-Phe sample.

FTIR Analysis

In our ATR-FTIR analysis, we focused on the Amide region (1700–1500 cm^{-1}), which strongly correlates with amyloid fibrils, as reported by Sarroukh et al.[1] Here, we describe the distinct peaks observed in different samples and their corresponding conformations.

In the Amide I range (1700–1600 cm^{-1}), several β-sheet signatures were identified, consistent with previous findings.[1] The A-β sample exhibits a strong peak at 1630 cm^{-1}, a characteristic marker of amyloid fibrils. The dipeptide spectrum features a peak at 1685 cm^{-1}, signifying the presence of anti-parallel β-sheets (APB). The presence of APB suggests that oligomers coexist with mature fibril structures in the sample. Similarly, in the A-β spectrum, a small shoulder appears in the same region, indicating the presence of oligomers in this sample as well. The single amino acid samples show a peak at 1640 cm^{-1}, which is indicative of parallel β-sheets (PB).

The distinct peak at 1605 cm^{-1} is attributed to a C-C ring vibration, which is notably absent in the A-β polypeptide spectrum. This observation is consistent with findings in other polypeptides containing phenylalanine,[2] where the side chain vibrations do not prominently appear in FTIR spectra.

A peak in the Amide II region (1600–1500 cm^{-1}) exhibits a shift between the North and South directions of magnetization. This 30 cm^{-1} shift is observed in both the A-β polypeptide (from 1530 to 1560 cm^{-1}) and the dipeptide Phe-Phe (from 1550 to 1580 cm^{-1}) but is absent in the single amino acid. This suggests a decrease in anti-parallel β-sheets (APBs) as aggregation progresses.[3] There are likely both oligomers and mature fibrils in each sample.

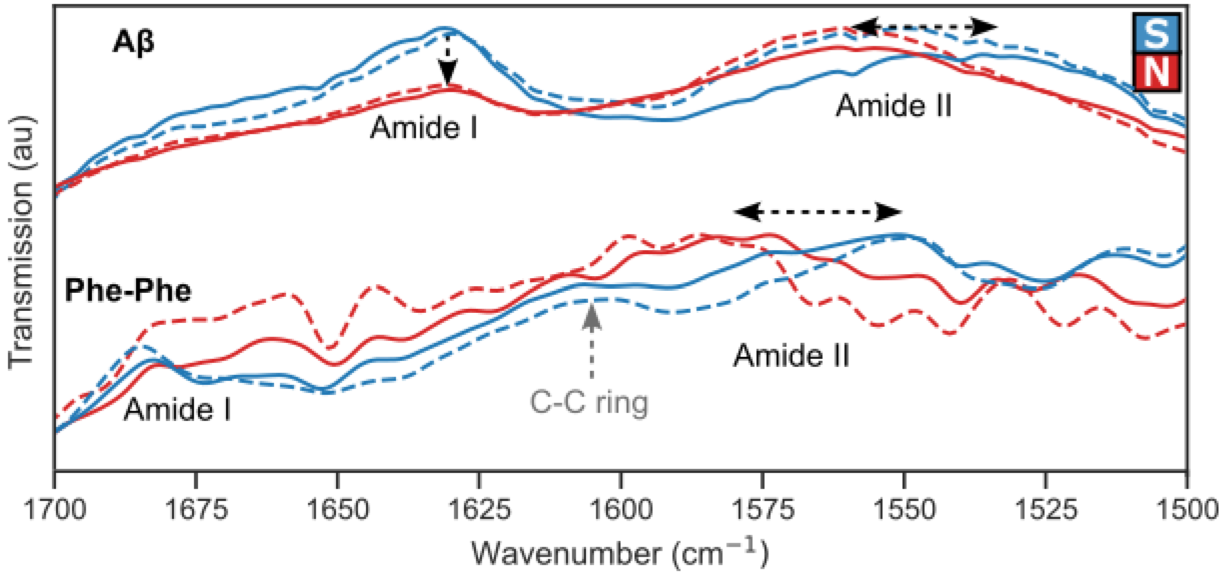

Figure S11: Repetitions of ATR-FTIR spectra for A-β polypeptide (top) and Phe-Phe dipeptide (bottom) assembled on South- (blue) or North- (red) magnetization orientation. The solid line is the spectrum taken for the samples of the main manuscript, while the dashed line is an additional spectrum from a different sample made under the same conditions.

Charge Termini Peptide

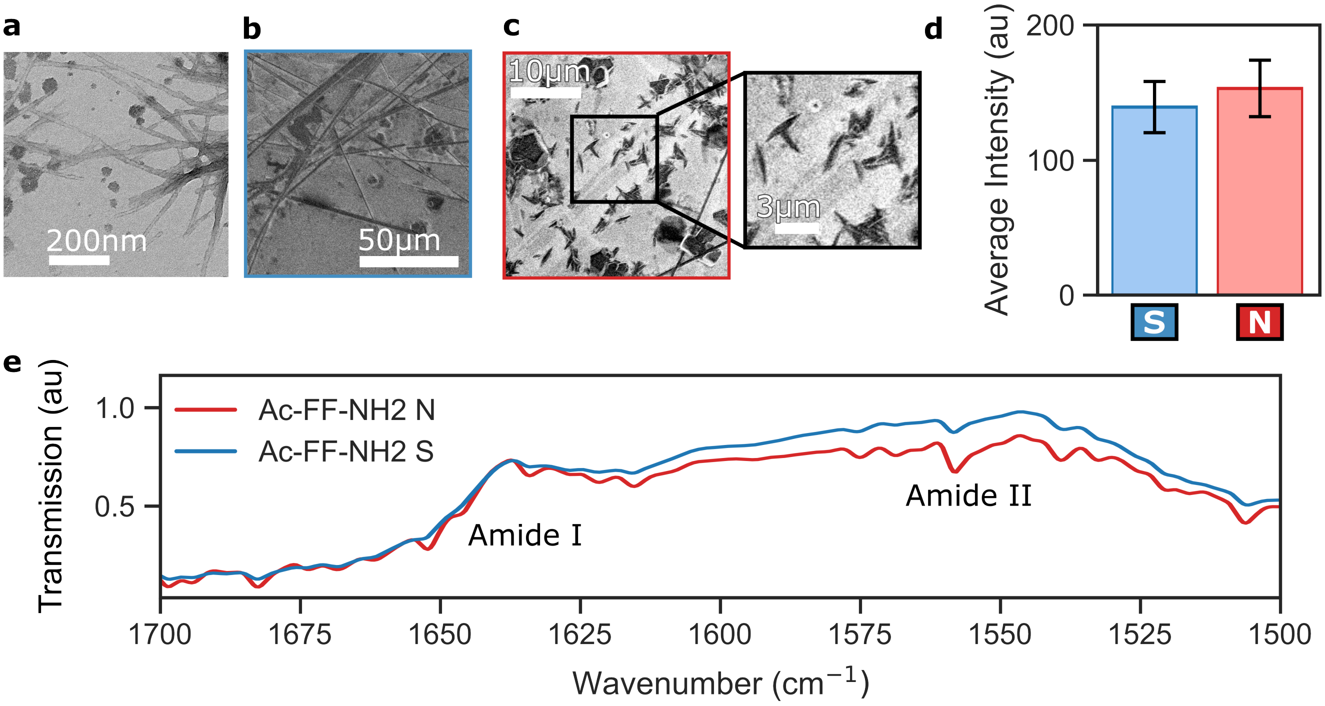

Figure S12: Characterization of Ac-Phe–Phe-NH_2. Negative staining TEM image showing fibrillar aggregates (**a**). SEM image of Ac-Phe–Phe-NH2 fibrils formed on a South-magnetized substrate (**b**) and on a North-magnetized substrate (**c**). Average fluorescence intensity of ThT dyed fibrils (**d**. FTIR spectra of Ac-Phe–Phe-NH2 fibrils formed on a South- or North-magnetized substrate.

To exclude the potential contribution of electrostatic interactions in the assembly process, we studied a modified, non-charged peptide analogue, Ac-Phe–Phe-NH_2, in which the N-terminal amine was acetylated and the C-terminal carboxyl was amidated. This modification reduces the peptide's dipole moment compared to the native form.[4] TEM imaging presented in Figure S12a confirmed that the analogue self-assembles into fibrillar structures (diameter 25nm and length 300nm). SEM images on North- and South- magnetized substrates are presented in Figure S12b,c. Significantly shorter fibrils were observed on the North side (>200μm vs. ∼3μm, similar to the behavior observed for the natural peptide. In addition, the assembly was more uniform on the South magnetized substrate. Fluorescence intensity measurements using a ThT dye (Figure S12d) and FTIR spectroscopy (Figure S12e)

showed no significant differences between fibrils formed on North- versus South-magnetized substrates. FTIR spectroscopy revealed an Amide I peak at 1640 cm^{-1} and an Amide II peak at 1545 cm^{-1} corresponding to the known β-sheet signatures as described in Reches et al.[4]

Our results show that while the peptide analogue forms similar fibrillar morphologies, its structural behavior differs from the native peptide. This suggests that electrostatic interactions may play a role in the assembly process and that these interactions could be influenced by spin effects.

Dynamic Assembly Growth

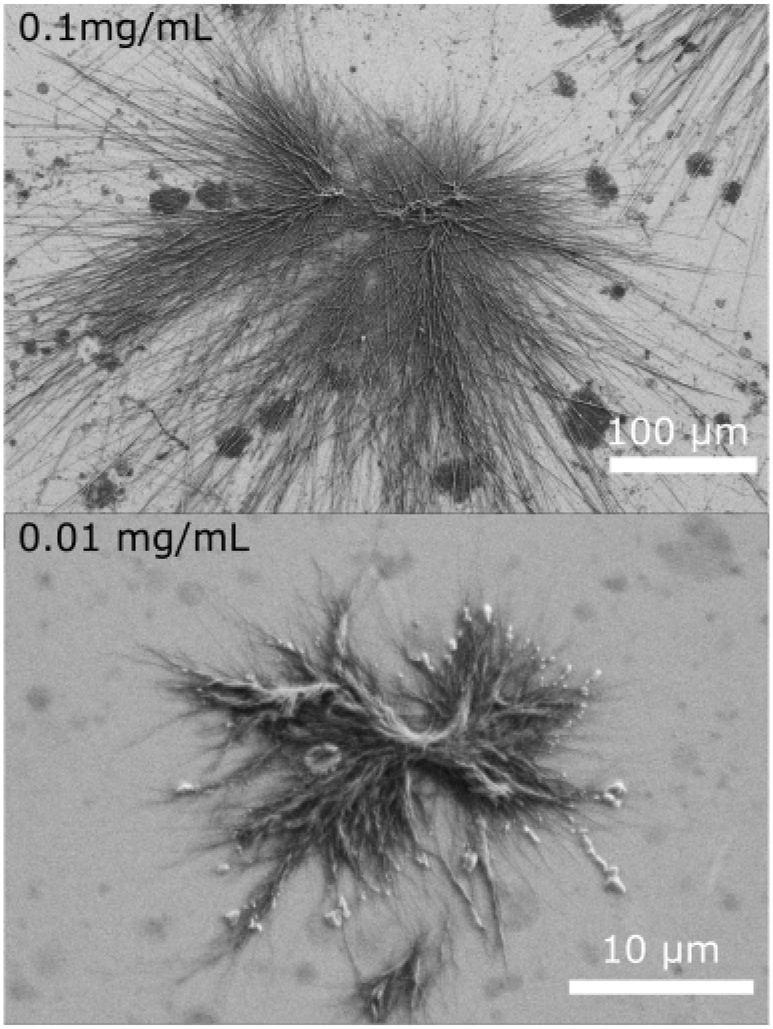

Figure S13: The nucleation dynamics of Ac-Phe2-NH2 peptide analogs were followed following the aggregation at different concentrations. Even at completely different concentrations (**top** - 0.1 mg/mL; **bottom** - 0.01 mg/mL), the monomers assembled into fibrils of similar morphology. This is likely due to the drying dynamics, which increase concentrations significantly.

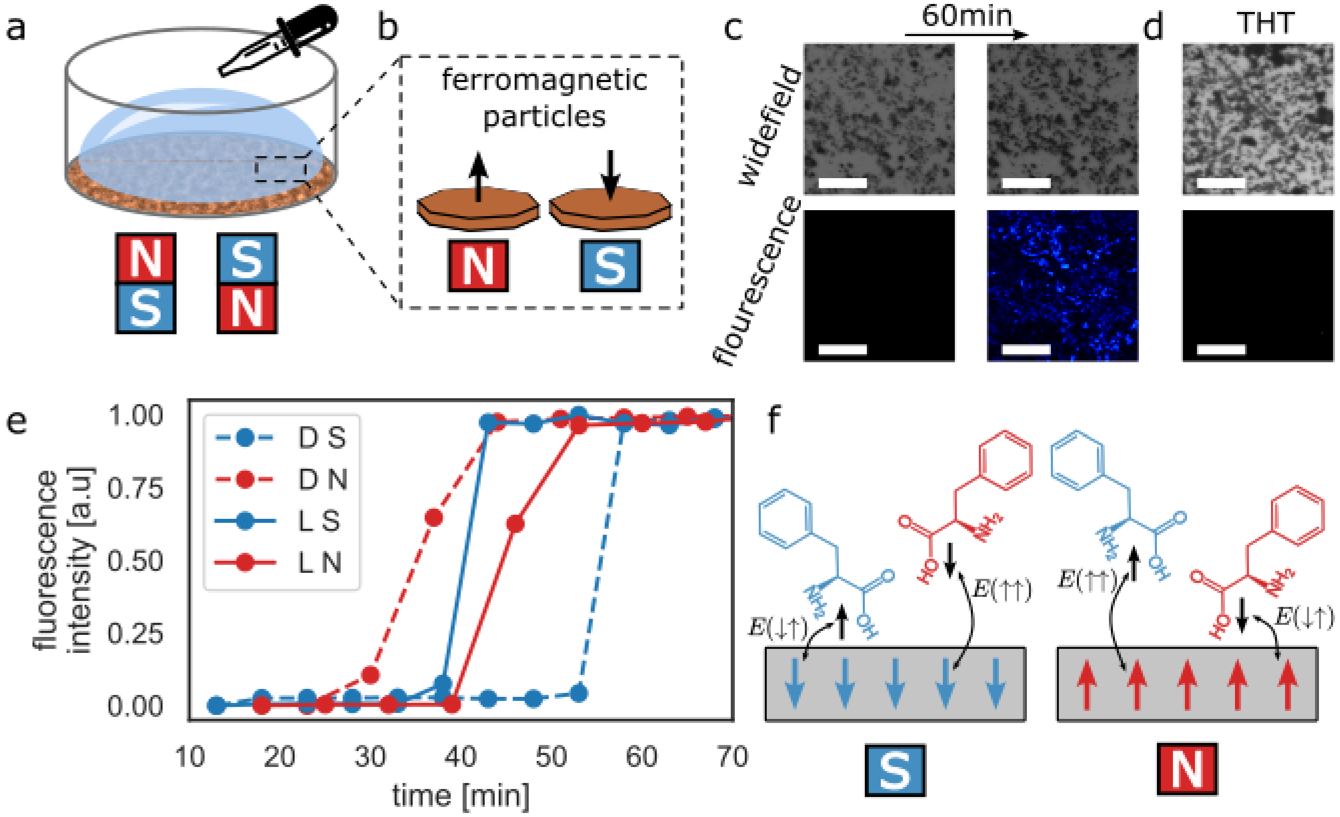

Figure S14: The dynamics of the Phe amino acid monomers were followed in real time by ThT assay using a confocal microscope. **a** a drop of Phe solution (preparation procedure is listed in the methods) is drop casted and assembled on a plate with ferromagnetic nano-platelets. **b** The nano-platelets are magnetized either up or down using a 0.5T magnet. The ferromagnetic nanoparticles were platelet shaped BaFe11.5In0.5O18 (In-doped barium hexaferrite) dispersed in water with an average thickness of 5 nm and a diameter range of 10–70 nm. They create small clusters that act as nucleation sites. **c** The assembly of the monomers is followed both by fluorescence and optical confocal microscopy every 5-7 minutes over 2 hours. After around 40 minutes ThT fluorescence appears, indicating fibrillar assembly. **d** A ThT reference without the monomers showed no fluorescence. **e** normalized fluorescence intensity of L-Phe (solid) and D-Phe (dashed) under North (red) and South (blue) magnetization orientations. A faster nucleation happens whenever the chirality and magnetization orientation are preferred (L and South, D and North). **f** This is due to parallel/ anti-parallel spin configurations between the monomer's electrons and the spin-polarized surface electrons. The anti-parallel (singlet-like) state is thermodynamically favored.

Non-Magnetic Substrates

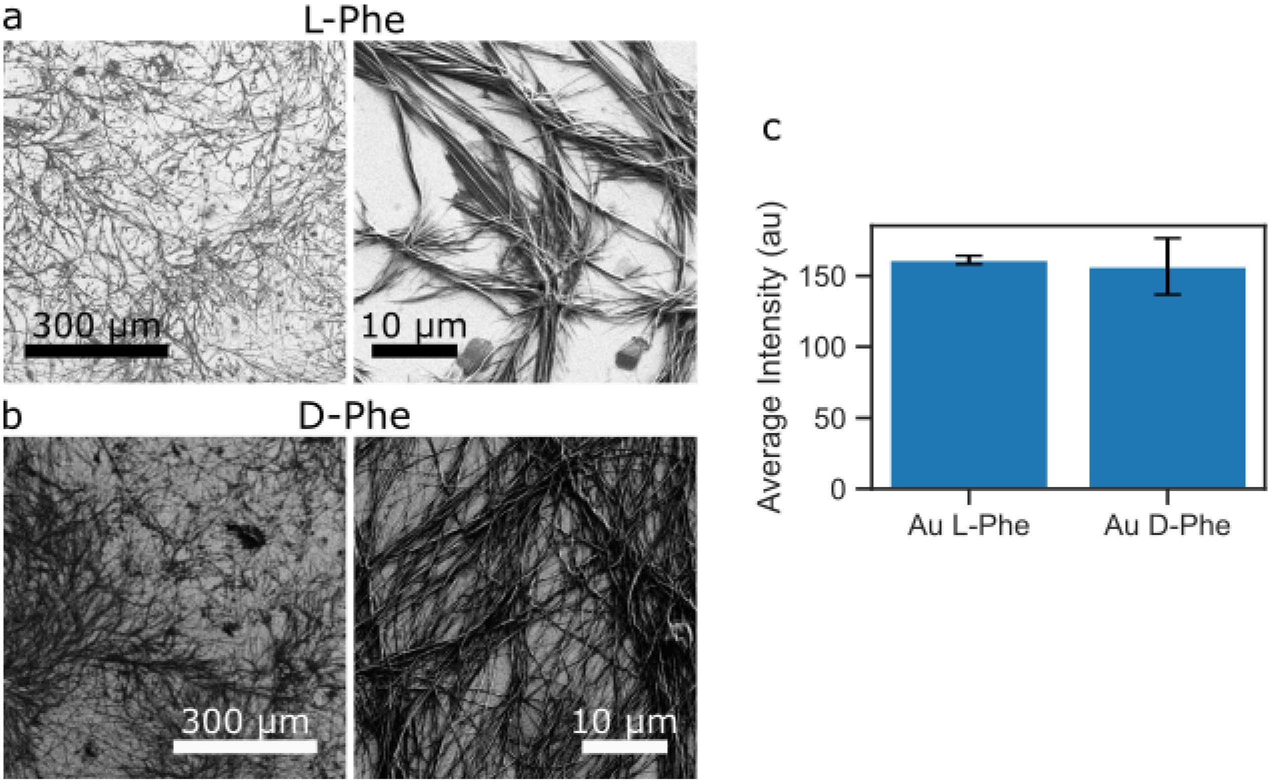

Figure S15: Control experiments on non-magnetic, non-polarizable substrates. **a,b** SEM images of fibrils formed by L-Phe (top) and D-Phe (bottom) on a sputtered Si/Cr(5 nm)/Au(50 nm) substrate, shown at two different magnifications. The morphology and spatial distribution appear similar for both enantiomers. **c** Averaged ThT fluorescence intensity of the same samples, showing no significant difference between L- and D-Phe. These results confirm that the differences observed on magnetized substrates are not present on non-magnetic controls, supporting a spin-polarization-based mechanism.

References

1. Sarroukh, R.; Goormaghtigh, E.; Ruysschaert, J.-M.; Raussens, V. ATR-FTIR: A "rejuvenated" tool to investigate amyloid proteins. *Biochimica et Biophysica Acta (BBA)-Biomembranes* **2013**, *1828*, 2328–2338.

2. Barth, A. The infrared absorption of amino acid side chains. *Progress in biophysics and molecular biology* **2000**, *74*, 141–173.

3. Sarroukh, R.; Cerf, E.; Derclaye, S.; Dufrêne, Y. F.; Goormaghtigh, E.; Ruysschaert, J.-M.; Raussens, V. Transformation of amyloid β (1–40) oligomers into fibrils is characterized by a major change in secondary structure. *Cellular and Molecular Life Sciences* **2011**, *68*, 1429–1438.

4. Reches, M.; Gazit, E. Self-assembly of peptide nanotubes and amyloid-like structures by charged-termini-capped diphenylalanine peptide analogues. *Israel journal of chemistry* **2005**, *45*, 363–371.
